# Cap-assisted hemoclip application with forward-viewing endoscope for hemorrhage induced by endoscopic sphincterotomy: a prospective case series study

**DOI:** 10.1186/s12876-015-0367-2

**Published:** 2015-10-15

**Authors:** Feng Liu, Guang-Yong Wang, Zhao-Shen Li

**Affiliations:** 1 Department of Gastroenterology, Changhai Hospital, Second Military Medical University, 168 Changhai Road, Shanghai, 200433 China; 2 Department of Gastroenterology, 411 Hospital of PLA, 15 Dongjiangwan Road, Shanghai, 200081 China

**Keywords:** Cap-assisted endoscopic hemoclip, Endoscopic sphincterotomy, Hemorrhage

## Abstract

**Background:**

Endoscopic sphincterotomy (ES) is a therapeutic technique developed as an advanced application of endoscopic retrograde cholangiopancreatography (ERCP). An important adverse event associated with this procedure is hemorrhage, which may sometimes be uncontrollable. We sought to examine whether cap-assisted hemoclip application is effective in controlling ES–induced hemorrhage.

**Methods:**

In this prospective study, we investigated the outcomes in 10 patients who had uncontrolled ES–induced hemorrhage and were treated by cap-assisted application of hemoclip with a forward-viewing endoscope.

**Results:**

Nine of the 10 investigated patients were successfully treated using the cap-assisted hemoclip technique with forward-viewing endoscope, yielding a success rate of 90 %. The patient with hemorrhage non-responsive to hemoclipping required catheter embolization of the bleeding artery after its identification by digital subtraction angiography. One of the 10 patients developed mild pancreatitis after the procedure, but was successfully managed conservatively.

**Conclusions:**

Cap-assisted hemoclip application with a forward-viewing endoscope appears to be an effective therapeutic modality for achieving hemostasis in cases of ES–induced hemorrhage, without the occurrence of any severe adverse events; we believe that this method should be considered as an option in the management of ES–induced hemorrhage.

## Background

Endoscopic retrograde cholangiopancreatography (ERCP) is routinely used in the diagnosis of conditions involving the upper gastrointestinal tract, including the bile ducts, pancreas, and gallbladder. Subsequently, the application of ERCP was extended to include endoscopic sphincterotomy (ES) with a view to manage the detected abnormalities, without the need for invasive surgery. Although recent improvements in the ES technique have markedly enhanced the safety of ES, the procedure is still associated with several adverse events [1–6]. Among all the potential risks associated with ES, risk of bleeding is a critical one and could be life threatening [7]. Reports indicate that 2–12 % of patients undergoing ES develop bleeding [8], with approximately 0.2 % of the patients having severe bleeding after the procedure [6]. Generally, most of the hemorrhage occurs during the procedure, although delayed bleeding could also occur several hours, or even days, after the procedure [7]. Further, while minor oozing is relatively common and usually self-limiting, brisk, pulsatile, or continued bleeding requires immediate management with aggressive hemostatic measures.

Conventionally, hemostasis management after ES comprises epinephrine (1:10,000) injection, compression, and diathermy. Epinephrine stops bleeding mainly via localized vessel contraction and compression. Submucosal epinephrine (1:10,000) injection is commonly used to control acute bleeding after ES and has a high success rate. However, the rate of re-bleeding is high, at 11–28.5 % [9]. Therefore, the effectiveness of epinephrine injection in treating severe, acute, and delayed bleeding after ES is not clear at present. In addition, epinephrine can cause severe adverse effects, especially those related to cardiac activity. Therefore, care should be taken during the management of patients with suspected or confirmed coronary heart disease or irregular heartbeat [10]. Diathermy is another treatment method used for acute or delayed bleeding after ES; it encompasses electrocoagulation and argon plasma coagulation (APC). Heating probes or bipolar probes are commonly used in electrocoagulation, which may lead to adverse events, including necrosis and perforation; the rate of re-bleeding after electrocoagulation is as high as 25 %. In addition, duct damage resulting from electrocoagulation could lead to acute pancreatitis. On the other hand, in APC, a jet of ionized argon gas is directed at the bleeding lesion via the endoscope, resulting in coagulation. As a procedure with minimal carbonization and vaporization, APC is effective in arresting hemorrhage from multiple points. However, APC is not as effective as epinephrine injection or hemostatic titanium clips in controlling arterial hemorrhage [11]. Fully covered metallic biliary stents have also been applied in the management of ES–induced hemorrhage and have afforded good outcomes [12]. However, several factors, including high cost, requirement of repeat ERCP for the removal of the metallic biliary stent, and high shift rate, limit the widespread application of this method in endoscopic hemostasis. In some cases, e.g., in cases of arterial hemorrhage, all of the abovementioned treatment methods fail to effectively control bleeding.

The use of the endoscopic hemostatic clip in the management of hemorrhage in the digestive tract was first described by Hayashi et al., and has gained widespread popularity [13, 14]. Lin et al. reported the application of hemoclip under duodenoscopic guidance in the treatment of 6 cases of severe ES–induced hemorrhage with a success rate of 88.8 %. In their study, 2 hemoclips were applied in each case, with the average number of hemoclips dropping off being 0.5 [15]. However, the plastic sheath of the duodenoscope may kink when passing over the elevator at the bottom of the instrument channel, leading to failure of clip deployment. This technically challenging aspect of the use of endoscopic clips has limited their application in the treatment of ES–induced hemorrhage [15, 16].

The cap-assisted hemoclip along with forward-viewing endoscope is widely used in the treatment of digestive tract bleeding induced by endoscopic mucosal resection (EMR) and endoscopic submucosal dissection (ESD) and appears to be effective, especially in treating arterial bleeding [17, 18]. Thus far, no study on post–ES management of hemostasis has been reported via the cap-assisted hemoclip with forward-viewing endoscope, although this endoscope is more convenient to use than the duodenoscope. Therefore, we aimed to evaluate the safety and effectiveness of cap-assisted hemoclip application with the forward-viewing endoscope in the treatment of uncontrolled ES–induced bleeding in this prospective case study.

## Methods

### Study design and patients

**Fig. 1:**
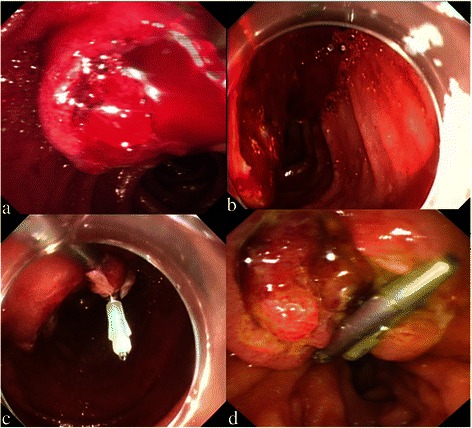
**a** Duodenoscope show uncontrollable bleeding after ES in a patient with choledocholithiasis; **b** Identification of the bleeding site using transparent cap-assisted forward-viewing endoscope; **c** Hemostasis with hemoclips under transparent cap-assisted viewing with forward-viewing endoscope; **d** After 4 days, the duodenoscope shows that the hemoclips are still located in the papillary area and bleeding is absent

Between April 2013 and April 2014, all 843 patients presenting to our endoscopic department with uncontrolled bleeding during or after ERCP and ES at Changhai hospital were prospectively enrolled in this study. Patients who had previously undergone ES and/or those having post-surgical anatomical changes were excluded. Among the remaining patients, 33 developed post–ES bleeding necessitating treatment and 11 of them had uncontrolled bleeding (Fig. 1). In one of the 11 cases, Billroth II surgery was required, and therefore, this case was excluded from further analysis. Thus, 10 patients with uncontrolled post–ES hemorrhage bleeding were included in this study. The characteristics of these patients are provided in Table 1.

**Table 1:** Clinical features of the patients

Age	Sex	Indications	Comorbidities	NSAID	Hb (g/L)	PLT (10^9)	INR	AMS (U/l) before surgery	AMS (U/l) after surgery	Transfusion (unit)
64	M	Choledocholithiasis	N	N	110	210	1.2	78	189	N
75	M	Choledocholithiasis	N	N	152	150	1	58	108	N
70	M	Choledocholithiasis diverticulum	Diabetes	Y	120	273	0.9	50	1692	4
74	M	Choledocholithiasis Suppurative cholangitis	Hypertension	Y	127	89	1.7	66	207	1
67	M	Choledocholithiasis	Diabetes, Gallstones	N	158	164	1.2	56	611	N
56	M	Choledocholithiasis diverticulum	N	N	124	162	1.3	142	210	N
				N						
59	F	Intraductal papillary mucinous neoplasm	N	N	89	267	1.4	89	105	1 (before surgery)
55	M	Choledocholithiasis diverticulum	Hypertension	N	130	269	1.2	59	110	2
				N						
60	M	Cholangiocarcinoma	N	N	110	100	1	64	85	N
70	M	Choledocholithiasis	N	N	98	126	1.5	78	148	N

Uncontrolled post–ES bleeding was defined as continued bleeding despite conventional treatment measures such as epinephrine (1:10,000) injection, compression for more than 5 min, and argon plasma coagulation [13]. The time to endoscopic hemostasis was defined as the total time from the insertion of the forward-viewing endoscope until the stoppage of bleeding. Acute bleeding during ES was defined as bleeding observed after incision of the papilla. Delayed ES–induced bleeding was defined as bleeding observed with a few hours to days after the ES procedure. Post-procedural adverse events were defined as adverse events related to the application of the cap-assisted hemoclip in ERCP/ES, including obstructive jaundice, acute cholangitis, and acute pancreatitis. Acute pancreatitis was defined as per the criteria specified previously [4]. The study protocol was approved by the ethical committee of Changhai hospital. Informed consent was obtained from all the patients before the start of the procedure.

### Procedure of endoscopic hemostasis

The procedure was performed using a forward-viewing endoscope (GIF-Q260J Olympus, Tokyo, Japan) with a front-end transparent cap (distal attachment with rim, MH-593 Olympus, Tokyo, Japan); the endoscope was advanced into the descending part of duodenum, close to the major duodenal papilla. The blood clots were then removed to expose the points of bleeding, and pressure was applied on these spots using the edge of the transparent cap and the clip (HX-600-135, Olympus; or M00522610, Boston-Resolution), and the clip was placed as close as possible to the point to avoid damage to the duct. After gentle suctioning via the transparent cap to centralize the lesion and the surrounding tissue, the bleeding vessel was clipped. All the ERCP procedures were performed by three physicians with extensive experience in ERCP (>200 cases in a year for more than 8 years).

### Data collection

Data were collected from each patient for the following parameters: patient demographics; comorbidities, including congenital and acquired hemostatic disorders; indication for the initial ERCP; treatment administered; hemostasis treatment techniques; time to hemostasis; treatment outcomes; and procedural adverse events.

## Results

The median age of the 10 enrolled patients was 66 years (range: 55 to75 years), and 90 % of the patients were men. Among the patients, eight had choledocholithiasis; 1 each had cholangiocarcinoma, bile duct papilloma, and cholangitis; and three had diverticula in the parapapillary region. Two patients had been taking aspirin, which was discontinued 5 days before the procedure. One patient had anemia that was corrected by transfusion of one unit of erythrocyte suspension before the operation. None of the patients had any evidence of bleeding diathesis.

All 10 patients underwent cap-assisted hemoclip placement with forward-viewing endoscope to arrest the bleeding (Table 2). The average length of the incision made on the papillary sphincter was 6.6 mm (range: 5–10 mm). Two patients had papillary balloon dilation during the operation. Nine of the patients had acute bleeding, while one had delayed bleeding. In seven cases, the bleeding occurred on the left side of the incision; in two cases, on the right side; and in one case, on both sides. Three patients required transfusion (range: 1–4 units). Further, three patients required plastic biliary stent implantation, while none required pancreatic stent implantation.

**Table 2:** Treatment outcomes

Age	Sex	Treatment		Bleeding occurrence		Bleeding site	No. of hemoclip (No. failed)	Time to hemostasis (min)	Outcome	Biliary stent	Adverse event
		ES (mm)	Balloon dilation (mm)	Acute	Delayed						
64	M	Y (5)	Y (8)	Y	-	Left	1 (0)	2	Success	y	N/A
75	M	Y (5)	-	Y	-	Left	1 (0)	24	Success	y	N/A
70	M	Y (5)	-	Y	-	Both sides	12 (2)	61	Fail	n	pancreatitis
74	M	Y (8)	-	Y	-	Left	4 (1)	7	Success	n	N/A
67	M	Y (10)	-	Y	-	Right	2 (0)	21	Success	y	N/A
56	M	Y (8)	-	Y	-	Left	2 (0)	14	Success	n	N/A
59	F	Y (8)	Y (10)	-	Y	Left	3 (0)	7	Success	n	N/A
55	M	Y (5)	-	Y	-	Left	1 (0)	20	Success	n	N/A
60	M	Y (5)	-	Y	-	Right	2 (0)	9	Success	n	N/A
70	M	Y (7)	-	Y	-	Left	2 (0)	9	Success	n	N/A

The vascular origins of the hemorrhage were identified using the forward-viewing endoscope, and hemoclips were applied to arrest the bleeding (Fig. 1). The average number of clips used was 3 (range 1–12), and the average number of clips dropping off was 0.3 (0–2). The average bleeding time was 17.4 min (2–61 min). The success rate of hemostasis was 90 %. In the only case in which the hemoclip failed to establish hemostasis, spurt bleeding was identified at 3, 6, and 12 points close to the incision lines. In this case, all the other bleeding controls methods attempted, including cold saline spray, epinephrine (1:10,000) injection, compression, and APC, failed. Additionally, 12 clips were applied (2 dropped off), but bleeding could not be arrested. Digital subtraction angiography (DSA) identified the posterior pancreatic duodenal artery as the source of the bleeding, and catheter embolization was subsequently performed to arrest the bleeding. This patient developed mild pancreatitis and was managed conservatively for the same. The patient did not develop any other adverse events related to clip application.

## Discussion

Traditionally, angiographic embolization or surgery is required when conventional methods of hemostasis management fail to arrest bleeding. These methods may, however, lead to clot formation in adjacent vessels and intestinal mucosal necrosis. The most important limitation to the surgical management of ES–induced bleeding is the underlying poor general health condition of the patients. Localized edema often causes enlarged lesions. In the extreme cases, retroperitoneal hematoma occurs with the shrinkage of the vessels underneath the mucosa, making them difficult to identify for suturing. The operative mortality rate in such cases is as high as 9 % [19]. Recent advances in non-surgical techniques have reduced the percentage of cases requiring surgical control of bleeding from 3 to 0.1 %. Further, the mortality rate associated with non-surgical approaches decreased from 1 % to below 0.1 % [20]. Thus, the development of non-surgical alternative approaches would be beneficial in the management of ES–induced hemorrhage.

Since ERCP is performed via a duodenoscope, normal hemostasis in cases of ERCP/ES–induced hemorrhage is generally achieved by duodenoscope-assisted surgery. Conventional strategies are usually effective in the management of ES–induced hemorrhage that is not severe. However, in some cases, most of the conventional methods fail to arrest the bleeding. Temporary placement of a covered self-expandable biliary stents across the biliary orifice is another treatment option, but it is limited by high cost, high shift ratio, and adverse events such as acute cholecystitis. Hemoclips are being extensively used to arrest bleeding, but when used with a duodenoscope, there is risk of developing bile duct obstruction and pancreatitis due to closure of the bile duct with clips; and there is technically challenging nature, only a few studies have been conducted thus far on using endoscopic hemostatic clips for achieving hemostasis in ES–induced hemorrhage [15, 16]. The technical problem is the difficulty in closing the clip with the duodenoscope when the elevator is fully elevated, with a short-route position of the endoscope; this is because of the tension created by the elevator on the clipping device. The designs and features of the new endoclips are evolving. For example, unlike older versions of the clips, the Instinct clips possess combined abilities to rotate, reopen, and reposition repeatedly, even when used with the duodenoscope. However, if the rotation ability of the instinct clip through the duodenoscope is good, the duodenoscope elevator must be in a half-locked position and not in the full-locked position [21]. Of course, this problem may be overcome by using a semi-long route position of the endoscope and with the elevator slightly released. However, in cases of massive bleeding, the visibility in the operative field is not adequate to identify the location of the bleeder, making it very difficult to clip with the duodenoscope. The placement of endoclips using side-viewing endoscopes is technically more difficult and results in misfiring more often than when used with forward-viewing endoscopes.

Cap-assisted forward-viewing endoscopy has been frequently used in endoscopic submucosal dissection or endoscopic mucosal resection for the treatment of superficial tumors within the digestive tract [22, 23]. The transparent cap increases the outer diameter and enhances the friction at the distal end of the endoscope, thereby improving the stability of the scope within the tube. This allows for better visualization of the lesion site, even at a distance from the mucosa. Additionally, it could be used to negotiate the mucosal folds that overhang the bleeding sites and improve visualization. Furthermore, the transparent cap would help counter the acute angle formed after intestinal anastomosis [24, 25]. Considering these points, we tested the feasibility of cap-assisted hemoclip application with the forward-viewing endoscope in the treatment of uncontrollable ES–induced bleeding.

We investigated 10 cases of uncontrollable ES–induced bleeding managed with cap-assisted forward-viewing endoscopy and successfully controlled the hemorrhage by identifying the bleeding sources and deploying the hemoclips in 90 % of the enrolled cases. The number of hemoclips dropping off was relatively low, and the time to hemostasis was short (Table 2).

We use forward-viewing endoscope (GIF-Q260J Olympus, Tokyo, Japan) with water jet function. We noticed that it was very useful for the hemostasis procedure. The water jet function is useful for identification of the bleeding point and establishment of hemostasis by washing out the extravasated blood, especially in cases of massive bleeding. However, the duodenoscope does not offer these advantages. Therefore, we think this is another reason justifying the use of the forward-viewing endoscope.

Bleeding sources at the right side of the incision are associated with potential risk of damage to the pancreatic duct when applying the hemoclips since the duct opens to the right side of the incision. However, it is unclear whether hemoclips can cause damage to the opening of the pancreatic duct and lead to pancreatitis. In two of our cases, the sources of bleeding were located on the right side of the incision. The excessive bleeding, incision on the papillary sphincter, and endoscopic balloon dilation make it difficult to determine the position of the opening of the pancreatic duct. However, even without locating the opening of the pancreatic duct, no adverse events, such as acute pancreatitis, were noted after hemostasis was established in our cases. If the ES–induced bleeding is not stopped effectively, patients have to undergo the additional procedures of DSA and surgery, which increases morbidity, cost, and risk of mortality from severe bleeding. Therefore, we recommend that the establishment of hemostasis and bleeding control be prioritized over the identification of the opening of the pancreatic duct, in cases where the latter is difficult. Appropriate postoperative care should be administered, depending on whether there are any adverse events, such as acute pancreatitis.

The major duodenal papilla receives it blood supply from the superior pancreaticoduodenal artery, inferior pancreaticoduodenal artery, and the communicating artery between these arteries [26]. Most of the arterial supply is distributed at anterior superior quadrant and posterior inferior quadrant. About 4 % of the patients have an abnormal branch of the inferior pancreaticoduodenal artery at the region of the incision, which can lead to severe bleeding after the papilla is incised. In seven of the 10 cases in this study, the bleeding sources were located on the left side of the incision, which may be related to the presence of the branch of the inferior pancreaticoduodenal artery.

Acute ES–induced bleeding has been associated with several risk factors, including hard stones, diverticula in parapapillary region, and accidental puncturing of a vessel by the needle-knife [27]. Delayed ES–induced bleeding, on the other hand, has been associated with coagulation abnormalities, heparin therapy, non-steroidal anti-inflammatory drug use, chronic liver and kidney dysfunction, hypertension, and ischemic heart disease [9]. The use of anticoagulants and antiplatelet medicine alone has not been reported as the sole risk factor [2]. In fact, the European Society for Gastrointestinal Endoscopy recommends that the use of aspirin and other non-steroidal anti-inflammatory drugs be continued, especially for patients with high risk of thromboembolism, since no obvious correlation was noted between these medications and the possibility of ES–induced bleeding [28]. In this study, two patients had been receiving oral aspirin and one each had anemia, reduced platelet count, and reduced international normalized ratio. One of the limitations of this study is that the small sample population in this study did not permit the determination of whether the use of anticoagulants and antiplatelet medicines was a risk factor for ES–induced bleeding.

## Conclusion

In conclusion, this is the first study on hemoclip application under cap-assisted forward-viewing endoscopic cover in the management of uncontrollable ES–induced bleeding. Our study revealed that this method was effective in controlling the bleeding, especially when the bleeding sources were along the incision, and that the method was relatively safe with minimal and mild adverse events. Further investigations in a larger population from multiple centers are necessary to confirm our findings.
